# Enhanced Heavy Metal Removal from Acid Mine Drainage Wastewater Using Double-Oxidized Multiwalled Carbon Nanotubes

**DOI:** 10.3390/molecules25010111

**Published:** 2019-12-27

**Authors:** Carolina Rodríguez, Eduardo Leiva

**Affiliations:** 1 Departamento de Ingeniería Hidráulica y Ambiental, Pontificia Universidad Católica de Chile, Avenida Vicuña Mackenna 4860, Macul, Santiago 7820436, Chile; cnrodriguez@uc.cl; 2 Departamento de Química Inorgánica, Facultad de Química, Pontificia Universidad Católica de Chile, Avenida Vicuña Mackenna 4860, Macul, Santiago 7820436, Chile

**Keywords:** multiwalled carbon nanotubes, oxidation, adsorption, heavy metal removal, acid mine drainage

## Abstract

Due to the unique properties of carbon nanotubes (CNTs), they have attracted great research attention as an emergent technology in many applications including water and wastewater treatment. However, raw CNTs have few functional groups, which limits their use in heavy metal removal. Nevertheless, their removal properties can be improved by oxidation processes that modify its surface. In this study, we assessed the capacity of oxidized and double-oxidized multiwalled carbon nanotubes (MWCNTs) to remove heavy metals ions from acidic solutions. The MWCNTs were tested for copper (Cu), manganese (Mn), and zinc (Zn) removal, which showed an increment of 79%, 78%, and 48%, respectively, with double-oxidized MWCNTs compared to oxidized MWCNTs. Moreover, the increase in pH improved the sorption capacity for all the tested metals, which indicates that the sorption potential is strongly dependent on the pH. The kinetic adsorption process for three metals can be described well with a pseudo-second-order kinetic model. Additionally, in multimetallic waters, the sorption capacity decreases due to the competition between metals, and it was more evident in the removal of Zn, while Cu was less affected. Besides, XPS analysis showed an increase in oxygen-containing groups on the MWCNTs surface after oxidation. Finally, these analyses showed that the chemical interactions between heavy metals and oxygen-containing groups are the main removal mechanism. Overall, these results contribute to a better understanding of the potential use of CNTs for water treatment.

## 1. Introduction

Water scarcity has become a major problem worldwide. Nowadays, population growth, urbanization, pollution, and climate change are some of the main factors responsible for water scarcity [1,2,3]. In particular, Chile has a variable climate and, consequently, a diverse industrial activity along it [4], i.e., mining activity is very common in the central and northern regions of Chile. The mining industry consumes around 7.2% of all water used in Chile and represents more than 50% of the water used in northern regions [4,5,6]. Besides, mining activity results in widespread pollution related to acid generation and heavy metal release known as acid mine drainage (AMD), which adversely affects the quality of surface and ground water resources [7,8]. Thus, new alternatives to improve the water availability are becoming increasingly necessary.

AMD is characterized by a low pH and a high concentration of heavy metals, such as iron (Fe), copper (Cu), manganese (Mn), and zinc (Zn), among others [7,8]. Commonly, treatment systems for AMD are classified as passive and active methods [9]. Passive treatments are mainly used in abandoned mines and include constructed wetlands, bioreactors, and chemical treatments [9,10]. On the other hand, the active treatment approach encompasses techniques such as adsorption, ion exchange, and membrane technology [9,11]. Several sorbent media have been tested for the removal of AMD contaminants (i.e., sulfate and heavy metals), which are made up of materials such as activated carbon (AC), granular activated carbon (GAC), and powdered activated carbon (PAC), which differ in their particle sizes [12]. Thus, there is a growing interest in the development of more efficient sorbent media.

In recent years, nanotechnology has become an emergent technology for many applications, including water treatment [3]. Nanomaterials are very interesting with regards to their applicability on water purification processes, mainly due to their unique properties, such as their high specific surface area, and its discontinuous properties [3,13,14]. One of the novel materials in this field are carbon nanotubes (CNT), composed of cylindrical graphite sheets arranged in a tubular structure [2,3,14,15,16]. There are two types of CNTs: single-walled carbon nanotubes (SWCNTs) that consists of a single shell of graphene; and multiwalled carbon nanotubes (MWCNTs), composed of multiple layers of graphene sheets [14,15]. In addition, CNTs form aggregated pores that provide a high external surface area [17]. CNTs and surface modifications of it have been tested for the removal of heavy metals through adsorption and have shown good results [15,18,19,20]. In general, it has been reported that raw CNTs have few functional groups, so their sorption capacity is very limited [19,21,22,23]. The main chemical treatments tested to increase the content of the functional groups are oxidation with different methods, and functionalization with amino groups and magnetite, among others [15,24,25].

Additionally, other nanomaterials like graphene-based sorbents have been studied for the removal of heavy metals. Between these graphene functionalized with pentaerythritol (a hydroxyl-abundant molecule) for the adsorption of rare earth elements [26]; or a bio-adsorbent synthesized by functionalization of persimmon tannin with graphene oxide for the adsorption of gold (Au), silver (Ag), and palladium (Pd) [27] have shown good results. Likewise, a Pb(II) imprinted magnetic bio-sorbent has been tested for the removal of lead (Pb) [28]; and a magnetic bio-sorbent hydrogel beads, prepared based on modified biopolymer gum tragacanth (GT) and graphene oxide were successfully used for the removal of Pb and Cu [29]. These studies show the wide variety of treatments and functionalizations for graphene-based sorbents, as well as the wide range of elements that can be removed with them.

The chemical interaction between metal ions and surface functional groups of CNTs is the primary sorption mechanism [30,31]. This interaction allows the sorption of divalent metals mostly [18,20,30,32]. Among the critical parameters that controls the metal adsorption onto the CNTs is the pH value. The point of zero charge (pH_PZC_) indicates the pH at which the sum of positives charges equals the sum of negatives charges [33]. Thus, under the pH_PZC_, the surface is positively charged and can attract anions. Conversely, above the pH_PZC_, the surface is negatively charged, and it attracts cations [33,34]. The pH_PZC_ is a fundamental parameter because it indicates the effectiveness of a sorbent depending on the pH of the water to be treated. CNTs have shown a good performance at low pH values for the adsorption of heavy metals, since they may have a pH_PZC_ of less than 5 [19,34,35]. This supports its use as a feasible alternative for the treatment of acidic waters. Similar technologies, such as biochar or activated carbon, commonly have pH_PZC_ greater than 7 [36,37,38], which limits its application for heavy metal-enriched waters with low pH.

Another key factor is the competition of metals for adsorption sites. When different metals coexist, competitive adsorption should be taken into consideration because the sorption/removal of an ion could be affected by the presence of other metals in the solution [39].

Several methods have been studied to improve the sorption capacity of MWCNTs for metal removal [15]. One of the most extended methods is chemical oxidation [18,19,40,41]. Chemical oxidation increases the defects on the CNTs surface, covering the ends and walls of the nanotubes with oxygen-containing groups such as carboxyl groups, hydroxyl groups, ether groups, among others [42,43]. The oxidation process depends on several factors, such as the oxidation time and the reflux temperature. In general, it has been reported that the average length of CNTs decreases significantly as the oxidation time and temperature increases [44,45,46]. However, other studies have shown that the CNTs structure does not change considerably when oxidation times are between 0.5 and 7 days [47]. Thus, the greatest differences are observed within the first hours of oxidation. The chemical functionalities added to the surface of the MWCNTs by oxidation processes create electrostatic stability that allows a colloidal dispersion [43]. This dispersion improves the surface contact between the MWCNTs and the surrounding medium, allowing more interactions between the functional groups and the ions present in the solution.

Given the current context of water scarcity and the growing demand for water and wastewater treatment technologies, the objective of this work was to explore the technological application of oxidized MWCNTs for the remediation of acidic waters contaminated with high concentrations of heavy metals. For this, modified MWCNTs were studied for the removal of Cu, Mn, and Zn. Successive oxidations were tested in order to reduce the pH_PZC_ and increase the removal efficiency at low pH. The adsorption capabilities were evaluated at different pH values, and the competitive adsorption of three heavy metal ions on oxidized and double-oxidized MWCNTs was studied. The focus of this study was on exploring the use of CNTs in complex waters that emulate real AMD waters. The relevance of this work lies in providing a better understanding of MWCNTs and their adsorption properties in multimetallic acidic solutions as an alternative for the removal of heavy metals from AMD runoffs. It is necessary to understand the metals adsorption/desorption mechanisms at low pH values for the development of applicable technologies for a real treatment system.

## 2. Results and Discussion

### 2.1. Characterization of MWCNTs

#### 2.1.1. Infrared (IR) Spectroscopy

**Figure 1:**
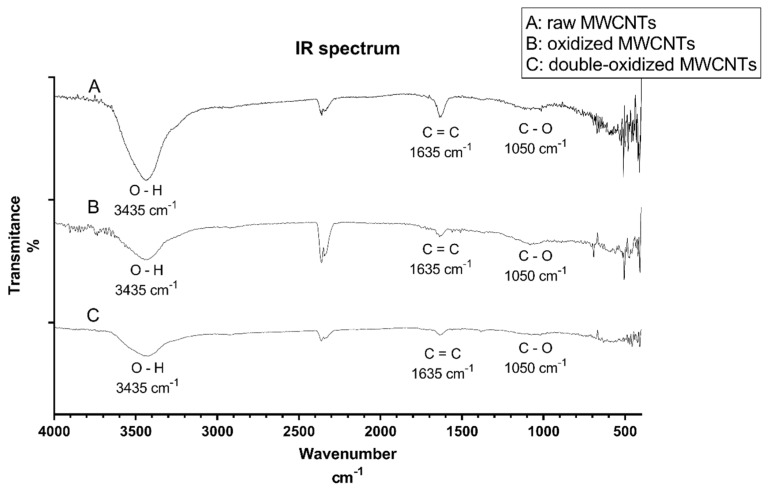
IR spectrum for A: raw, B: oxidized, and C: double-oxidized MWCNTs.

The infrared (IR) spectroscopy analysis was carried out to study the functional groups present on MWCNTs after oxidation processes. Figure 1 shows the IR spectrum of raw, oxidized, and double-oxidized MWCNTs. The three samples show the same three major signals: 3435 cm^−1^, 1635 cm^−1^, and 1050 cm^−1^. The peak of 3435 cm^−1^ indicates the O─H bond and is more intense in raw than in oxidized MWCNTs. Raw MWCNTs show an intense peak in 1635 cm^−1^ corresponding to the C=C bond in benzene rings [48]. It can be inferred that according to the intensity of the signal, raw MWCNTs have a higher amount of C=C bonds, and when MWCNTs were oxidized, these bonds were broken, resulting in the formation of new bonds [49]. The peak around 1050 cm^−1^ corresponds to C─O bond related to carboxyl groups introduced in the oxidation processes. The peaks near to 2300 cm^−1^ are present in all three samples and correspond to carbon dioxide, which is a typical signal of this analysis [48]. Appendix B shows the IR spectrum for samples of oxidized and double-oxidized MWCNTs after adsorption processes, peaks corresponding to O–H and C=C functional groups can be observed.

#### 2.1.2. Brunauer–Emmet–Teller Analysis (BET)

The BET analysis gives information on the surface properties and the sorption capacity for different types of MWCNTs. The BET surface area, pore volume, pore diameter, maximum adsorption capacity, and point of zero charge are summarized in Table 1. The BET surface area increased from 157.34 to 179.53 m^2^/g after the first oxidation step, but it decreased during the second oxidation showing an S_BET_ value of 105.54 m^2^/g. The decrease in the BET surface area in acid treated CNTs has been previously reported. This occurs mainly because the defective sites of the CNTs are occupied by the functional groups incorporated during the oxidation process [50,51]. Even so, these values are high compared to other values reported using different oxidation methods [30,52], which suggests a high sorption potential. The same behavior observed for S_BET_ values was observed for the maximum adsorption capacity, which increased from 36.15 to 41.25 cm^3^/g and then reduced to 24.25 cm^3^/g with double oxidation. However, it should be noted that the BET analysis gives information about the material potential, but in practice, the sorption effectiveness will depend on the amount and type of functional groups that are present in MWCNTs, as well as on the dispersion that they can reach in solution.

In addition, the point of zero charge of a surface is an essential factor in determining the metal adsorption effectiveness, because waters with a high metallic content generally have a low pH. Thus, the surface charge of MWCNTs can be estimated by pH_PZC_ values. Untreated nanotubes have a pH_PZC_ value of about 4.65, which means that for pH values above the pH_PZC_, the MWCNTs surface will be negatively charged, and therefore will have an affinity/potential for cations removal. Inversely, below this value, the surface will be positively charged, and the interaction with metals will be minimal [33,34]. Furthermore, the pH_PZC_ of oxidized MWCNTs is of about 3.70, and the value for double-oxidized MWCNTs is even lower. These pH_PZC_ values represent a decrease compared to raw MWCNTs. This suggests that the pH range at which oxidized and double-oxidized MWCNTs can remove metals is bigger compared to untreated MWCNTs. Successive oxidation steps partially opens the MWCNTs tips, which enhances the dispersion of MWCNTs and generates structural defects due to the attachment of oxygen-containing groups [53]. The presence of these strongly acidic groups can be evidenced by the great and sustained decrease in the pH_PZC_ values with successive oxidations [53,54]. Therefore, the decrease in pH_PZC_ values from 3.7 to 1.2 with double oxidation can promote a best interaction and removal of metals from acidic waters.

#### 2.1.3. Scanning Electron Microscopy (SEM)

**Figure 2:**
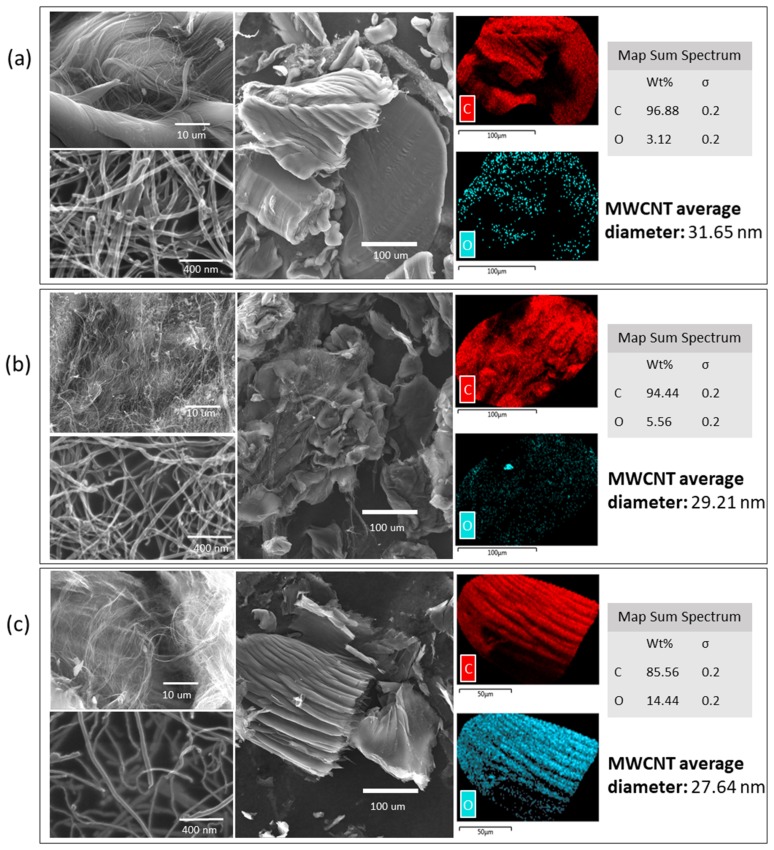
Field emission scanning electron microscopy and scanning electron microscopy coupled with energy-dispersive X-ray spectroscopy (SEM–EDX) of (**a**) raw, (**b**) oxidized, and (**c**) double-oxidized MWCNTs.

The morphology of raw and oxidized MWCNTs was investigated with scanning electron mcroscopy coupled with energy-dispersive X-ray spectroscopy (SEM–EDX), and field emission scanning electron microscopy (FESEM). SEM/FESEM images are presented in Figure 2. It is possible to observe morphological differences between the raw and oxidized MWCNTs. The raw MWCNTs have a homogeneous surface. After the oxidations processes, the nanotube surface is heterogeneous, and, with the double oxidation, the filaments looked thinner and were divided into smaller segments. Also, it is possible to observe an increase in the oxygen weight percentage (wt %) after the oxidation processes, which may be explained by the addition of new functional groups (e.g., hydroxyl and carbonyl) (Figure 2). Furthermore, the average diameter of the MWCNTs decreases with the oxidation processes, from a diameter of 31.65 nm for untreated MWCNTs to 29.21 nm and 27.64 nm for oxidized MWCNTs and double-oxidized MWCNTs, respectively. The reduction in diameter with successive oxidations indicates the removal of impurities such as metals and amorphous carbon, and sidewall MWCNTs damage resulting from oxidation [43].

In consequence, the MWCNTs characterization analysis showed that the main differences derived from the oxidation treatment involve the formation of new bonds due to the union of functional groups to the surface. These functional groups contain oxygen, which was confirmed by an increment in the content of this element. Moreover, the surface changed considerably due to the defects introduced during the oxidation, which could improve the adsorption potential of MWCNTs.

### 2.2. Adsorption Experiments

The adsorption experiments were performed with acidic-metallic solutions with varying concentrations of divalent metals (Cu^2+^, Mn^2+^, and Zn^2+^). The experiments are shown only for the oxidized and double-oxidized MWCNTs since raw MWCNTs showed a negligible removal capacity (data not shown). It has already been reported that raw MWCNTs have a negligible sorption capacity. Some studies present sorption values that do not exceed 1.6 mg/g [22,23].

Experimental data were fitted with Langmuir and Freundlich isotherm models. It can be seen that the Langmuir model has a better agreement with the observations compared to the Freundlich isotherm. Furthermore, we should recall that the Langmuir isotherm defines the equilibrium parameters of homogeneous surfaces and monolayer adsorption [55], while the Freundlich model describes a multilayer adsorption onto a heterogeneous surface [56].

**Figure 3:**
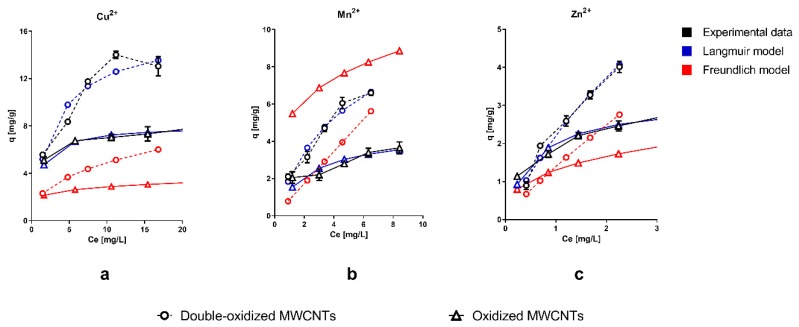
Adsorption isotherms of single ions of (**a**) Cu^2+^, (**b**) Mn^2+^, and (**c**) Zn^2+^ onto oxidized MWCNTs (continuous lines) and double-oxidized MWCNTs (dashed lines). Experimental data, Langmuir isotherm model, and Freundlich isotherm model are shown in black, blue, and red, respectively. Error bars of experimental data correspond to the standard deviation of the experiments made in triplicate. In some cases, the error bars are not observed because the standard deviation of the results is small.

Figure 3 shows that the sorption capacity significantly increased with the double oxidation (double-oxidized MWCNTs) for the three metals studied, compared with a single oxidation (oxidized MWCNTs). In the case of Cu^2+^, the maximum sorption capacity increased from 7.8 to 14 mg/g. These results imply an increment of 79% in the total removal within 14 h of contact time. For Mn^2+^ and Zn^2+^, the maximum sorption capacity increased from 3.7 to 6.6 mg/g and from 2.7 to 4.0 mg/g, which correspond to an increment of 78% and 48%, respectively. For all three metals, the best removal capacity was achieved with the double-oxidized MWCNTs, which confirms the improvement in the removal potential due to the double oxidation. Similar studies show variable sorption capacities, depending on the type of treatment used in the CNTs and the specific experimental conditions. Form previous studies, sorption capacities between 3.5–29 mg/g for Cu^2+^, 0.2–2.0 mg/g for Mn^2+^, and 0.27–58 mg/g Zn^2+^ have been reported for MWCNTs [15,19,20,30,57,58,59,60]. This strongly supports our statement that the oxidation process improves the sorption capacity of CNTs.

The major difference in the sorption capacities occurs at high metal concentrations, while at low concentrations, the difference is less significant. It has been reported that heavy metals are absorbed at specific sorption sites for low metal concentrations [61]. Furthermore, at low metal concentration, the competition for binding sites is lower [61]. Therefore, at low metal concentrations, the removal capacity should be similar for different nanotubes (oxidized and double-oxidized MWCNTs), which was consistent with our results. At higher metal concentrations, it is possible to observe the differences in the affinity of MWCNTs with the different metals studied. The good affinity of MWCNTs with Cu^2+^, compared to other metals, has been reported on several studies [35,62].

**Figure 4:**
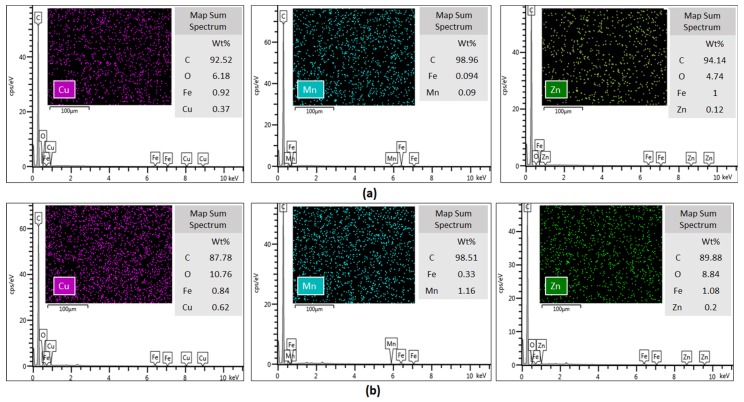
Energy-dispersive X-ray spectroscopy of (**a**) oxidized and (**b**) double-oxidized MWCNTs after the sorption experiments. The intensity of Cu, Mn, and Zn emission lines are shown in EDS spectrum, suggesting the stabilization of these metals onto the MWCNTs surfaces.

SEM images (Figure 4) confirm the hypothesis of metal stabilization on the surface of MWCNTs. Similarly to what we observed in the adsorption isotherms, the metal retention is greater for double-oxidized MWCNTs than for oxidized MWCNTs. Although the SEM-EDS analysis is not quantitative, we observed an increase in the metals weight percentage as determined by EDX analysis in double-oxidized MWCNTs compared with oxidized MWCNTs. The identified spectrum showed that there were 0.37% of Cu, 0.09% of Mn and 0.12% of Zn atoms on the surface of the double-oxidized MWCNTs, while on the surface of oxidized MWCNTs a lower weight percentage was detected with values of 0.62%, 1.16%, and 0.2% for Cu, Mn, and Zn, respectively. Although the examined area was very small compared to the total sample, we observed that the metals distribution on the surface was homogeneous. These results reinforce the idea that double-oxidized MWCNTs are better sorbents for divalent metals from acidic waters.

The metal sorption capacity has also been studied for other types of adsorbents. Recently, Soltani et al. [63] measured sorption capacities of about 7.30 mg/g for Cu using clay as the adsorbent, and of about 22 mg/g using polyaniline-clay. It should be noted that these concentrations are much higher than the ones studied in this work (between 50 and 1000 mg/L). Moreover, other adsorbents have been used successfully for the removal of various metals, such as the graphene oxide-maize amylopectin adsorbent that reached a sorption capacity of 30.56 mg/g for Cu and of 7.92 mg/g for Mn [64]. In addition, a new imprinted magnetic biosorbent reached a sorption capacity of 69.34 mg/g for lead [28].

### 2.3. Competitive Adsorption

**Figure 5:**
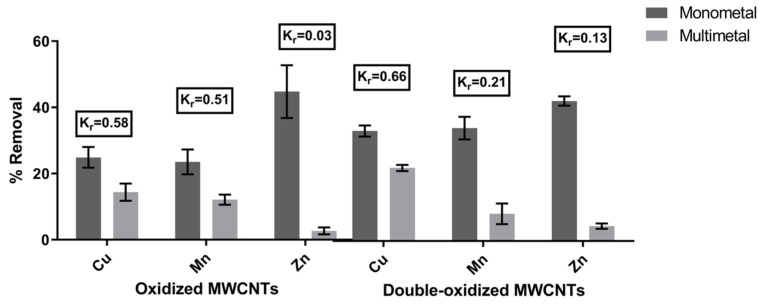
Comparison of the removal rates of the metals in monometal and multimetal solutions. The removal coefficient (K_r_) was calculated for both treatments. Error bars correspond to the standard deviation of the experiments made in triplicate.

The sorption experiments were carried out for the same metal concentration and pH value, using a monometallic and a multimetallic solution. The removal rates for each case are illustrated in Figure 5. In order to quantify the effect of competition between different metals in acidic waters, we calculated the removal coefficient (Kr). A metal with higher Kr implies a lower effect of competition on that metal in the presence of other metals. According to the results, the calculated values of Kr were 0.58 (Cu^2+^), 0.51 (Mn^2+^), and 0.03 (Zn^2+^) for oxidized MWCNTs and 0.66 (Cu^2+^), 0.21 (Mn^2+^), and 0.13 (Zn^2+^) for double-oxidized MWCNTs. Thus, Kr values for the studied metals in decreasing order are Cu > Mn > Zn, which means that the metal that is least affected by the presence of other metals is Cu, while the most affected by competition is Zn. Previously, Salam et al. [65] showed similar results in which MWCNTs had a better removal of Cu than Zn.

It can be seen that the effect of competition affects metals proportionally to their initial concentration in the solution (Cu: 20 mg/L, Mn: 6 mg/L, Zn: 3 mg/L). Therefore, the dominant factor for the preference between the different ions corresponds to the competition for the active sites of the adsorbent and the availability of ions in the aqueous solution [18]. This has been already demonstrated in several studies, where also the pH plays an important role in the ion’s competition. It has been reported that the increase in pH can improve the competition of some metals [18,19,65], i.e., Stafiej and Pyrzynska [19] showed that at pH above 6, Cu^2+^ greatly improves its competition against Mn^2+^. Hence, these results support the advantage of the double oxidation in enhancing the removal potential of the MWCNTs.

### 2.4. pH Effect on Adsorption Rate

**Figure 6:**
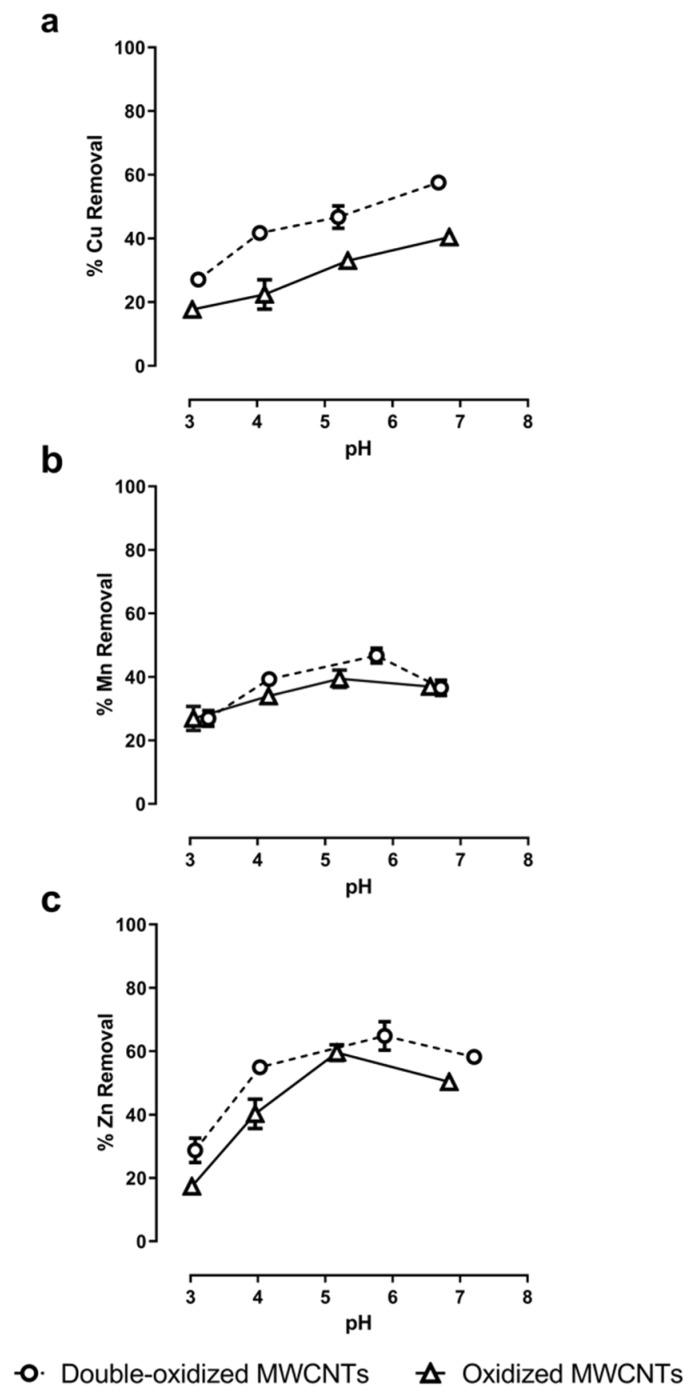
Effect of pH in the adsorption of (**a**) Cu^2+^, (**b**) Mn^2+^, and (**c**) Zn^2+^ onto oxidized MWCNTs (continuous lines) and double-oxidized MWCNTs (dashed lines). Error bars correspond to the standard deviation of the experiments made in triplicate. In some cases, the error bars are not observed because the standard deviation of the results is very small.

Sorption assays under different pH values were performed to evaluate the role of the solution’s pH in the adsorption rate. The pH values studied range from 3.0 to 7.0. The results show a direct relationship between the pH of the solution and the metal removal (Figure 6). In general, the sorption capacity increases with increasing pH. At very acidic pHs (3.0–4.0), removal rates were minimal for all three metals. This can be explained because, for pH values much lower than pH_PZC_, the surface of the MWCNTs will be more positive, and hence it will reduce its ability to interact with divalent cations. 

For Cu^2+^ ions, the removal rate increased from 18% to 40% with oxidized MWCNTs and from 27% to 58% with double-oxidized MWCNTs. The sorption rates increased steadily as the pH increased. These results are in accordance with what has been observed for Cu^2+^ removal with functionalized MWCNTs. Specifically, Zhang et al. [66] achieved an elimination rate close to 72% by increasing the pH up to 12. Likewise, the same effect was observed by Stafiej and Pyrzynska [19]. They found that at pH 9 the affinity order of the metal ions using CNTs as adsorbents was Cu(II) > Pb(II) > Co(II) > Zn(II) > Mn(II) [19].

In the case of Mn^2+^, the assays with oxidized MWCNTs show that sorption rates increased from 27% to 40% at a pH value around 5.2 and then it decreased to 37% at circumneutral pH. With double-oxidized MWCNTs, the removal rates increased from 27% to 47% at a pH value of around 5.5 and then decreased to 37% at a pH of 6.5. Both types of MWCNTs had a similar behavior for different pH values. The decrease in Mn removal for high values of pH has already been reported [67]. When the pH decreases, the surface of the MWCNTs becomes more positive due to the deposition of hydrogen ions on the surface. In contrast, when the pH increases, the surface becomes more negative, that is, it has a greater affinity to remove cations [33,67]. This behavior would explain what happens with the removal of Mn^2+^ up to pH ~5.5. From this value, a decrease in Mn removal is observed with the increase in pH, which could be related to the Mn speciation as a function of pH. Using PHREEQC (acronym of pH-Redox-Equilibrium-C + language program) speciation models (Appendix A, Table A1), we observed that at high pH (>6.0) the formation of manganese hydroxides (Mn(OH)_3_^−^) occurs, which can affect the removal process of Mn^2+^ because this anion is not removed by negatively charged surfaces. 

Finally, for Zn^2+^, the maximum sorption rates are reached at a pH close to 5.5, similar to Mn^2+^, reaching a removal of 60% with oxidized MWCNTs and of 65% with double-oxidized MWCNTs. Similarly, other studies have reported that the Zn^2+^ removal by CNTs becomes constant at pH~6.0, and then it decreases as the pH increases [68]. This behavior can be also explained by the increase in the formation of Zn hydroxides (Zn(OH)_3_^−^) as a function of pH (Appendix A, Table A1).

### 2.5. Kinetics Experiments

**Figure 7:**
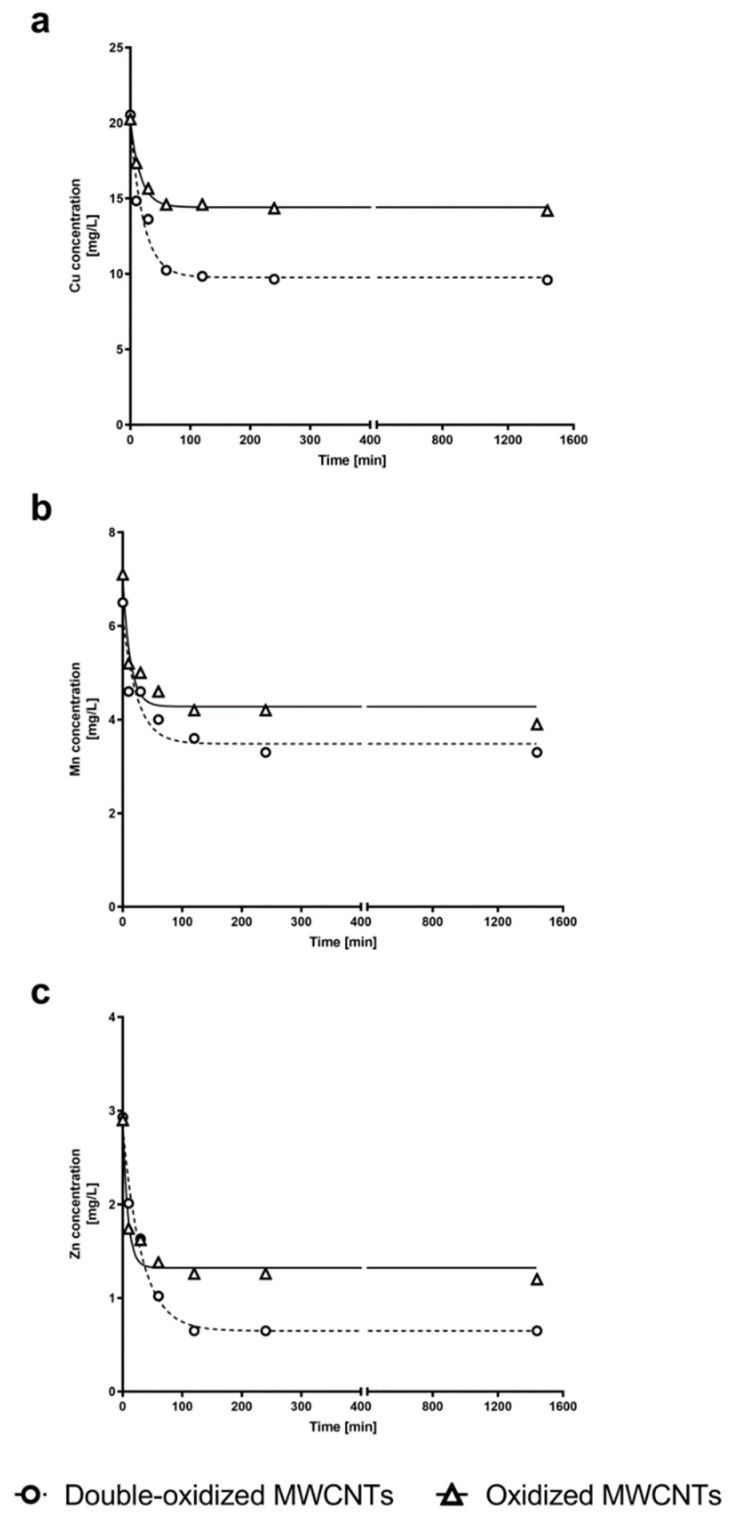
Concentration of (**a**) Cu^2+^, (**b**) Mn^2+^, and (**c**) Zn^2+^ in water solution at different times. The experimental data for Cu^2+^, Mn^2+^, and Zn^2+^ concentrations over time are shown for kinetics experiments with oxidized MWCNTs (triangle) and double-oxidized MWCNTs (circles). Fitting curves are shown for oxidized MWCNTs (continuous curves) and double-oxidized MWCNTs (dashed curves). Nonlinear regression was used for fitting model of kinetics adsorption experiments.

In order to study the adsorption kinetics of the three metals by oxidized and double-oxidized MWCNTs, kinetics experiments were performed. The experimental kinetic curves for oxidized and double-oxidized MWCNTs showed that the sorption of Cu^2+^, Mn^2+^, and Zn^2+^ rapidly increased within the first 10 min of contact time. The sorption continued to increase over time until it reached an equilibrium at approximately 2 h of contact time (Figure 7). Similar results have been reported for the adsorption of metals by different CNTs. In these cases the equilibrium was reached approximately between the first 30 and 120 min [69,70]. Li et al. [41] investigated the removal of Cu^2+^ and observed that the adsorption rate increased rapidly during the first 15 min and reached its equilibrium point at approximately 60 min.

We calculated the parameters of the model from the experimental data to describe the kinetic process. The parameters and the regression correlation coefficients (R^2^) are presented in Table 2. Both the theoretical equilibrium sorption capacity ($q_{e}$) model and the values of the correlation coefficient show that the pseudo-second-order model is better suited to reproduce the experimental data for the three metals studied (Cu^2+^, Mn^2+^, and Zn^2+^). These results support the idea that the sorption process is mainly controlled by the adsorption reaction at the liquid/solid interface at the adsorbent, and not by a diffusion-controlled process as the pseudo-first-order model would indicate [71].

### 2.6. Desorption Experiments

**Figure 8:**
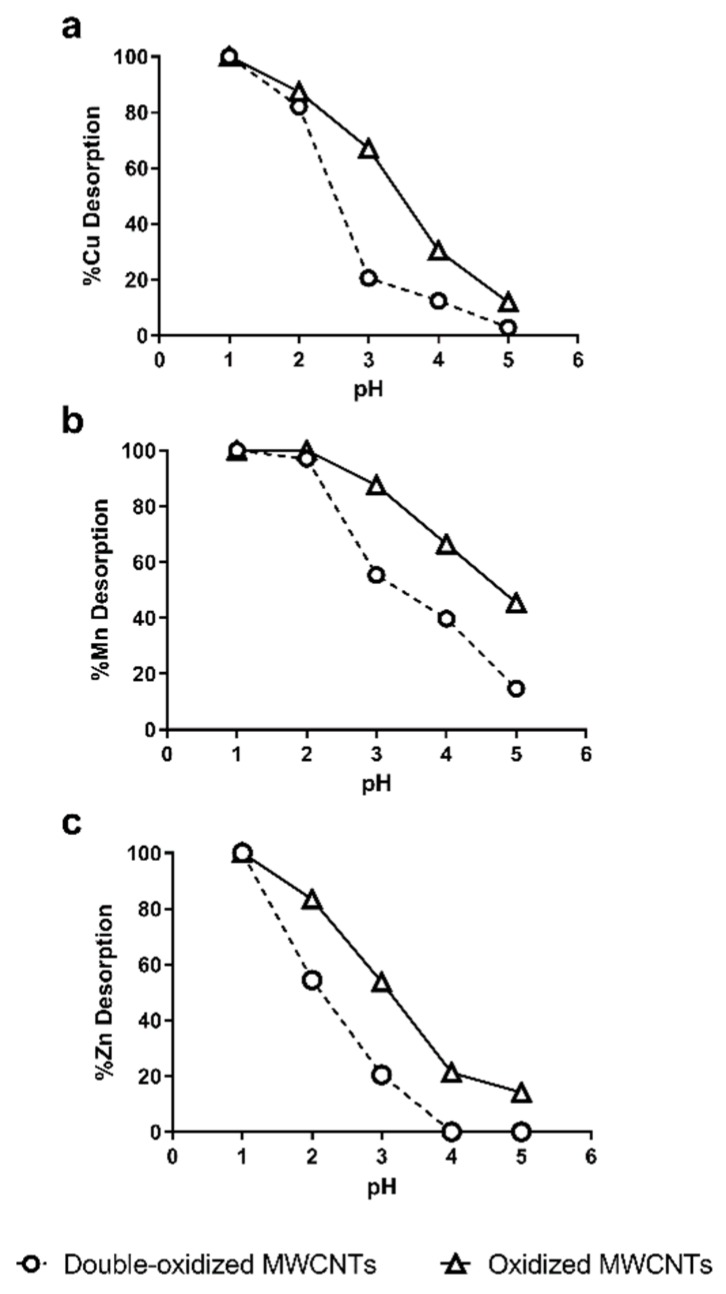
Effect of pH in the desorption of (**a**) Cu^2+^, (**b**) Mn^2+^, and (**c**) Zn^2+^ from oxidized MWCNTs (continuous lines) and double-oxidized MWCNTs (dashed lines).

The desorption of heavy metals was studied as a function of pH. The desorption process is important because the recycling and regeneration of the adsorbent are necessary to reduce operating costs [62]. On the other hand, this information also reports about the stability of adsorption in the treatment of acidic waters. Figure 8 shows the Cu^2+^, Mn^2+^, and Zn^2+^ recovered from oxidized and double-oxidized MWCNTs at pH values between 1 and 5.

It can be observed that for the three metals, the percentage of desorption at the same pHs was lower for double-oxidized MWCNTs than for oxidized ones. These results indicate that double-oxidized MWCNTs generate more stable chemical interactions at low pH, so they have a better sorption capacity for acidic waters, as it is also shown in Figure 6. However, both types of MWCNTs show almost a total desorption for a pH values below 2. Other studies with oxidized CNTs have shown similar behavior even with different metals. Liang et al. [72] used MWCNTs for the removal of Mn and obtained a desorption of ~80% at pH 4 and of 100% at pH 3. Moreover, Lu et al. [73] used SWCNTs and MWCNTs for the adsorption of Zn. In both cases, they obtained a 60% of desorption at a pH 3 and more than 90% at pH 1 [73]. For other metals, a desorption greater than 80% has been observed at higher pHs than those used obtained in this study, i.e., pH 4.5 for titanium and pH 3 for Pb^2+^ [70,74,75,76].

### 2.7. X-ray Photoelectron Spectrometry (XPS) Analysis

**Figure 9:**
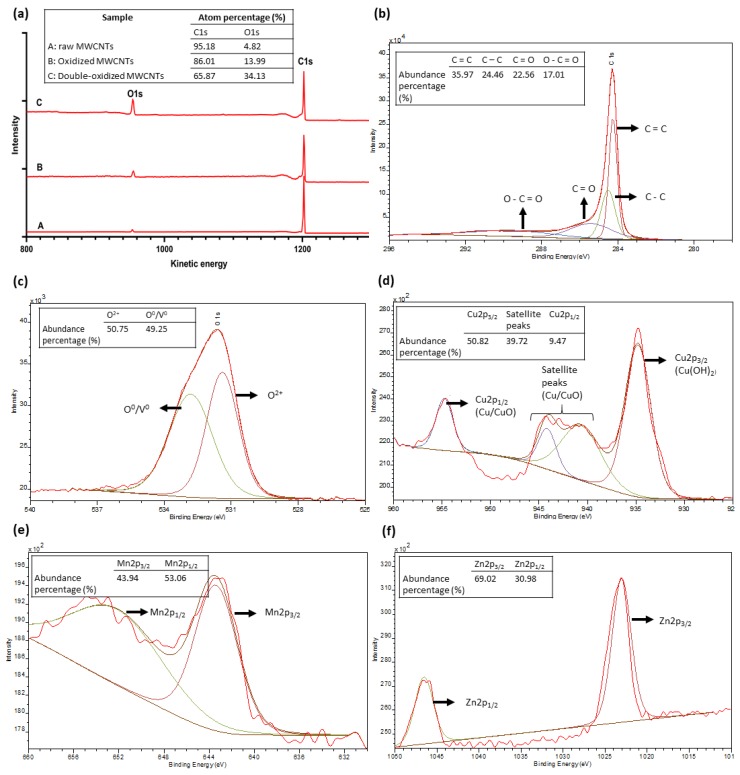
XPS spectra of (**a**) raw, oxidized, and double-oxidized MWCNTs; (**b**–**f**) high-resolution XPS C1s, O1s, Cu2p, Mn2p, and Zn2p narrow scan as function of electron binding energy.

The XPS analysis was used to quantify the surface elemental composition of MWCNTs and to understand the adsorption mechanism. Figure 9 summarizes the results of this analysis. Figure 9a shows the full spectrum for MWCNTs samples. The abundance percentages for the primary XPS region of carbon (C1s) and oxygen (O1s) are 95.18% and 4.82% for raw MWCNTs, 86.01% and 13.99% for oxidized MWCNTs, and 65.87 and 34.13% for double-oxidized MWCNTs, respectively. According to literature data, oxidized CNTs have oxygen percentages in the order of 8–12% in relation to carbon content [77,78,79]. On the other hand, the oxygen abundance in raw CNTs usually does not exceed 3% [77,78,80], which is consistent with the results obtained in this study. Thus, these results show a significant increase in oxygen content due to the formation of oxygen-containing functional groups. Other studies on the effect of oxygen-containing groups on the performance of adsorbents have been performed. A significant increase in the sorption capacity of biochar [81] and graphene oxides [82,83] was reported, with the increase in the content of oxygen-containing groups.

Figure 9b shows the deconvoluted spectra of C1s. The analysis shows that about 40% of carbon content corresponds to chemical bonds of functional groups that contain oxygen. On the other hand, Figure 9c, and d show the deconvolution of the spectra of O1s and Cu2p, respectively. The O1s peak can be divided into two parts. The first peak, around 531 eV corresponding to O^2−^, which interacts with Cu^2+^ to form chemical bonds, and another peak around 533 eV, attributed to surface absorbed O^0^ or oxygen vacancy (V^0^) [84,85,86]. The Cu spectrum shows a peak at 934.8 eV, representing the copper hydroxide (Cu(OH)_2_) [87]. This area represents about 50.82% of the total abundance. In addition, satellite peaks are observed, which according to the literature, is related to Cu^2+^/CuO [84,87]. Finally, a smaller peak corresponding to Cu2p_1/2_ is observed at 944.1 eV, which is also attributed to Cu^2+^/CuO [84,85]. Therefore, Cu can be found as superficial Cu^2+^, which may explain a physical removal of copper attached to the surface of the MWCNTs. However, Cu can be observed mostly as copper oxide/hydroxide, which demonstrates that the main removal mechanism corresponds to the chemical interaction between Cu^2+^ and the functional groups of oxidized MWCNTs. The Mn spectrum (Figure 9e) shows two major peaks, corresponding to Mn2p_1/2_ (~652 eV) and Mn2p_3/2_ (~643 eV). These peaks and their positions are typical of manganese oxides. In particular, the shape of the peak Mn2p_3/2_ and the absence of satellite zones around 647 eV, indicate that manganese is mostly found as manganese(III) oxide (Mn_2_O_3_) [88,89,90]. Likewise, the Zn spectrum (Figure 9f) also has two main peaks, corresponding to Zn2p_1/2_ (~1046 eV) and Zn2p_3/2_ (~1023 eV) that indicate the presence of zinc oxide (ZnO) [91,92]. Therefore, Cu, Mn, and Zn are forming oxides, which indicates that the main removal mechanism is the chemical interaction between metal ions and oxygen-containing groups, forming ionic bonds. This type of chemical bond between metallic and non-metallic atoms makes us conjecture that the main mechanism for removal is ionic exchange. This deduction was also obtained by some studies that investigated adsorption in CNTs [18,19,53].

The XPS analysis was complemented with desorption experiments as the pH value affects the speciation of the metals [93,94]. In the case of Cu, copper hydroxide is the dominant species for pH values greater than 7, and it decreases with the reduction of the pH value [95]. Finally, for pH values below ~2.5, Cu is fully dissolved as Cu^2+^, which is consistent with the desorption results shown in Figure 8 [95,96]. Furthermore, Mn and Zn are predominantly found as divalent species Mn^2+^ and Zn^2+^ at pH below 9 and 8, respectively [97].

## 3. Materials and Methods

### 3.1. MWCNT Oxidation

The MWCNTs, commercially acquired from NanoTechLabs, Inc. (Yadkinville, NC, USA) (purity > 95%, avg. length = 100 µm), were oxidized to introduce defects in their structure and increasing the number of functional groups present in it. 

#### 3.1.1. Oxidized MWCNTs

For this carboxylation method, 500 mg of MWCNTs were oxidized with 250 mL of 65% nitric acid (HNO_3_) by refluxing at 120 °C for 4 h. Then, the solution was filtered using a 0.45 µm membrane filter. Subsequently, the MWCNTs were washed with deionized water until reaching a neutral pH. The obtained solid was dried at 60 °C for 12 h. 

#### 3.1.2. Double-Oxidized MWCNTs

This method consists of two consecutive oxidations. First, MWCNTs are oxidized with 65% HNO_3_ as described above. Subsequently, a second oxidation was carried out using a proportion of nitric acid and sulfuric acid (H_2_SO_4_) [23]. For this, 500 mg of oxidized MWCNTs were treated with a (*v*/*v* 1:3) mixture of 65% HNO_3_ and 97% H_2_SO_4_. The mixture was sonicated at 40–50 °C for 3 h in an ultrasonic bath (Isolab Laborgerate GmbH, Wertheim, Germany). Then, the resulting solution was filtered using a 0.45 µm membrane filter and was washed with deionized water until the pH was neutral. The obtained solid was dried at 60 °C for 12 h.

**Figure 10:**
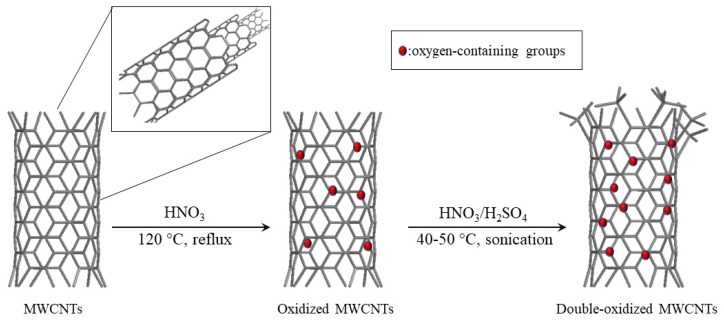
Schematic diagram for MWCNT oxidation.

The schematic diagram of both oxidation processes is presented in Figure 10.

### 3.2. MWCNTs Characterization

The raw, oxidized, and double-oxidized MWCNTs were characterized by infrared (IR) spectroscopy using the potassium bromide pastille method; Brunauer–Emmet–Teller analysis (BET) (Micromeritics Instruments Corp., Norcross, GA, USA); and scanning electron microscopy (SEM) (JSM-IT300LV, JEOL Ltd., Tokyo, Japan) coupled with energy-dispersive X-ray spectroscopy (Oxford Instruments, HighWycombe, UK) (SEM–EDX). Additionally, field emission scanning electron microscopy (FESEM) (Quanta FEG 250, FEI Company, Hillsboro, OR, USA) was used to obtain higher resolution images and to calculate the average diameter of the MWCNTs.

### 3.3. Point of Zero Charge (PZC)

To measure the PZC, we prepared 0.01 M solutions in 10 mL tubes at different pH values (2 to 10) adjusted with NaOH 0.1 M and HCl 0.1 M [33]. Then, 10 mg of raw, oxidized and double-oxidized MWCNTs were added to these solutions and shaken at 350 rpm at room temperature for 24 h. The final pH was measured using a pH meter (PHC301, HACH). Initial pH was plotted against final pH, and the intersection of this curve with the line that represents pH initial = pH final, determine the PZC value.

### 3.4. Adsorption Experiments

Adsorption experiments were performed for raw, oxidized, and double-oxidized MWCNTs. For this, 20 mg of MWCNTs were mixed with 30 mL of metal ion solution. The concentration was increased for each metal: Cu (5 to 25 mg/L), Mn (2 to 10 mg/L), and Zn (1 to 5 mg/L). The solution with MWCNTs was shaken for 14 h at 350 rpm. The resulting metal concentration was measured colorimetrically in a Hach spectrophotometer DR/2010. The experiment was performed in triplicate to obtain the average and standard deviation of the data.

### 3.5. Adsorption Isotherms

Experimental results were compared with Langmuir and Freundlich isothermal models. The sorption capacity q (mg/g sorbent) was obtained using the equation

(1) $$q=\frac{\left(C_{0}{-C}_{e}\right)V}{m}$$

where:

– $C_{0}$: initial concentration of metal in aqueous solution (mg/L).
– $C_{e}$: equilibrium concentration of metal in aqueous solution (mg/L).
– V: total volume of solution.
– m: the mass of sorbent.

#### 3.5.1. Langmuir Model

The sorption capacity q (mg/g sorbent) was obtained with the equation [98]

(2) $$q=\frac{q_{L}K_{L}C_{e}}{{1+K}_{L}\mathrm{Ce}}$$

where:

– $q_{L}$: amount of adsorption corresponding to a monolayer coverage.
– $K_{L}$: Langmuir constant related to the energy of adsorption.
– $C_{e}$: equilibrium concentration of metal in aqueous solution (mg/L).

#### 3.5.2. Freundlich Model

The sorption capacity q (mg/g sorbent) was determined as [98]:

(3) $${q=K}_{F}C_{e}^{\frac{1}{n}}$$

where:

– $K_{F}$ and n are Freundlich constants related to adsorption capacity and adsorption intensity, respectively.
– $C_{e}$: equilibrium concentration of metal in aqueous solution (mg/L).

### 3.6. Competitive Adsorption Experiments with Synthetic Acid Mine Drainage Waters

To study the interference between metals, adsorption experiments, as described in Section 2.4, were carried out using a synthetic acid mine drainage water. The synthetic water was designed, based on samples from typical rivers impacted by AMD in the central-northern zone of Chile. The synthetic water was prepared with distilled water, and it contained (per liter): 20 mg of Cu^+2^ (added as CuSO_4_·5H_2_O); 6 mg of Mn^+2^ (added as MnSO_4_·H_2_O); and 3 mg of Zn^+2^ (added as ZnSO_4_·7H_2_O). The pH was adjusted to 4.2 with 0.5 M HCl. Also, the removal coefficient (K_r_) was calculated to determine the metal sorption changes for monometallic and multimetallic solutions.

(4) $$K_{r}=\frac{\%\;\mathrm{Removal}\;\mathrm{in}\;\mathrm{multimetallic}\;\mathrm{water}}{\%\;\mathrm{Removal}\;\mathrm{in}\;\mathrm{monometallic}\;\mathrm{water}}$$

The experiment was performed in triplicate to obtain the average and standard deviation of the data.

### 3.7. Effect of pH

Adsorption experiments were repeated at fixed pH values according to the PZC results. For oxidized and double-oxidized MWCNTs, assays were carried out at pH 3, 4, 5.5, and 7 approximately. The experiment was performed in triplicate to calculate a mean and standard deviation for the data. 

### 3.8. Kinetic Studies

The kinetic studies were carried out using 20 mg of MWCNTs and 30 mL of metal solution. The metal concentration for the assays was the same used in interference experiments. The samples were taken at 10 min, 30 min, 1 h, 2 h, 4 h, and 24 h. The removal rate was represented as a function of time. The kinetic process was investigated according to a pseudo-first-order and pseudo-second-order kinetic equations. The pseudo-first-order kinetic model is presented by Lagergen as [99,100]:

(5) $$\log\left(q_{e}{-q}_{t}\right)=\log q_{e}-\frac{k_{1}}{2.303}t$$

where $q_{e}$ and $q_{t}$ are the adsorption capacity in mg/g at equilibrium and at time t, respectively, and k_1_ is the constant of first-order adsorption in min^−1^. The pseudo-second-order rate equation is expressed as [100]:

(6) $$\frac{t}{q_{t}}=\frac{1}{k_{2}\cdot q_{e}^{2}}+\frac{t}{q_{e}}$$

where $k_{2}$ is the constant of second-order adsorption in g mg^−1^ min^−1^.

### 3.9. Desorption Experiments

Adsorption experiments were carried out for oxidized and double-oxidized MWCNTs using monometallic solutions of Cu, Mn, and Zn. Assays were conducted by shaking 20 mg of MWCNTs with 30 mL of solution at room temperature until reach equilibrium. Then, the solution was filtered, and the filtrate was transferred to 30 mL of deionized water adjusted at different pH values (1 to 5) and was shaken for 14 h. The percentage of desorption was calculated using the equation

(7) $$\%\;\mathrm{Desorption}=\frac{\mathrm{amount}\;\mathrm{of}\;\mathrm{metal}\;\mathrm{released}\;\mathrm{to}\;\mathrm{solution}\;(\frac{\mathrm{mg}}{L})}{\mathrm{total}\;\mathrm{adsorbed}\;\mathrm{metal}\;(\frac{\mathrm{mg}}{L})}$$

### 3.10. X-ray Photoelectron Spectrometry (XPS) Analysis

The chemical composition of the MWCNTs surface, as well as the interaction with metals, was further characterized by X-ray photoelectron spectrometry (XPS, SPECS FlexMod, Berlin, Germany) equipped with PHOIBOS 150 1D-DLD analyzer and with monochromatic source Focus 500. Casa XPS software was used for detailed data processing.

## 4. Conclusions

In summary, our results showed that double-oxidized MWCNTs have a high capacity to remove metals from acidic waters. The oxidation treatment was useful to incorporate functional groups onto the MWCNTs surface. The chemical interactions between the functional groups and Cu^2+^, Mn^2+^, and Zn^2+^ were evidenced from the XPS analysis. This interaction may be the main removal mechanism. Indeed, the Cu^2+^ removal was more favored due its higher concentration in the acidic solution (simulated) compared with Mn^2+^ and Zn^2+^.

Double-oxidized MWCNTs were more efficient than oxidized MWCNTs in the removal of Cu^2+^, Mn^2+^, and Zn^2+^. SEM-EDX analyzes support this idea, because the metals weight percentage was higher in double-oxidized MWCNTs samples. Also, the metals were removed more efficiently at circumneutral values of pH, but Cu^2+^ tightly stuck to double-oxidized MWCNTs with a less desorption percentage compared to Mn^2+^ and Zn^2+^. Furthermore, kinetic experiments suggest that the pseudo-second-order model is a better representation of the Cu^2+^, Mn^2+^, and Zn^2+^ adsorption compared to the pseudo-first-order model.

In general, double-oxidized MWCNTs showed better performance in all cases, which supports the idea of its use as an alternative sorbent for metals from acidic waters. These findings contribute to a better understanding of the potential of MWCNTs for water treatment. 

## Figures and Tables

**Table 1:** Surface characteristics of the different types of MWCNTs studied.

Sample	S_BET_ (m^2^/g)	V_p_ (cm^3^/g)	D_p_ (nm)	Q_m_ (cm^3^/g)	pH_PZC_
Raw MWCNTs	157.34	1.50	34.30	36.15	4.65
Oxidized MWCNTs	179.53	1.25	25.59	41.25	3.70
Double-oxidized MWCNTs	105.54	0.98	31.73	24.25	1.20

S_BET_: BET surface area; V_p_: pore specific volume; D_p_: pore diameter; Q_m_: maximum adsorption capacity; pH_PZC_: point of zero charge.

**Table 2:** Kinetic adsorption parameters obtained using pseudo-first-order and pseudo-second-order models.

Sample	Metal	Initial Concentration (mg/L)	$q_{e}^{\exp}\;(\mathrm{mg}/g)$	Pseudo-First-Order			Pseudo-Second-Order		
				$k_{1}\;(\min^{-1})$	$q_{e1}\;(\mathrm{mg}/g)$	R^2^	$k_{2}\;(g/\mathrm{mg}\;\min)$	$q_{e2}\;(\mathrm{mg}/g)$	R^2^
Oxidized MWCNTs	Cu^2+^	20.25	9.08	0.0115	1.56	0.7878	0.0118	9.20	0.9994
	Mn^2+^	7.1	4.80	0.0067	1.26	0.7788	0.0231	4.54	0.9984
	Zn^2+^	2.9	2.55	0.0099	1.22	0.7612	0.0740	2.53	0.9994
Double-oxidized MWCNTs	Cu^2+^	20.53	16.34	0.0200	2.29	0.9188	0.0048	17.27	0.9971
	Mn^2+^	6.5	4.8	0.0035	1.21	0.2875	0.0110	5.10	0.9953
	Zn^2+^	2.93	3.44	0.0235	1.29	0.7886	0.0137	3.75	0.9957

**Figure A1:**
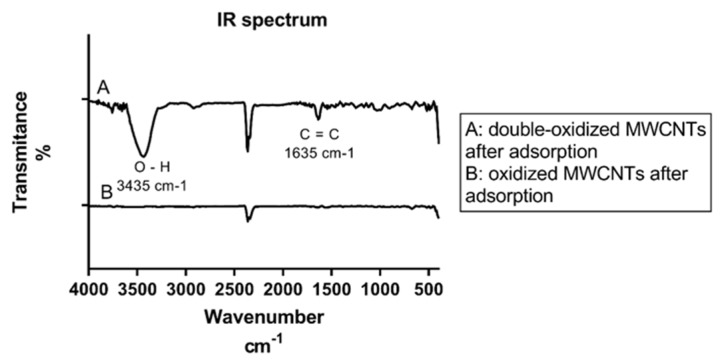
IR spectrum for A: double-oxidized MWCNTs and B: oxidized MWCNTs after adsorption experiments.

## Appendix A

**Table A1:** Molality of manganese and zinc hydroxides as function of pH. The speciation data corresponds to a simulation with PHREEQC using experimental concentrations of the analysis.

Phase	pH	Molality
Manganese hydroxide Mn(OH)_3_^−^	3.0	1.734 × 10^−30^
	4.0	1.795 × 10^−27^
	5.0	1.804 × 10^−24^
	6.0	1.805 × 10^−21^
	7.0	1.804 × 10^−18^
	8.0	1.801 × 10^−15^
Zinc hydroxide Zn(OH)_3_^−^	3.0	1.680 × 10^−24^
	4.0	1.753 × 10^−21^
	5.0	1.765 × 10^−18^
	6.0	1.765 × 10^−15^
	7.0	1.747 × 10^−12^
	8.0	1.444 × 10^−9^

## References

[1] Molinos-Senante M., Donoso G. (2016). Water scarcity and affordability in urban water pricing: A case study of Chile. Util. Policy.

[2] Adeleye A.S., Conway J.R., Garner K., Huang Y., Su Y., Keller A.A. (2016). Engineered nanomaterials for water treatment and remediation: Costs, benefits, and applicability. Chem. Eng. J..

[3] Gehrke I., Geiser A., Somborn-Schulz A. (2015). Innovations in nanotechnology for water treatment. Nanotechnol. Sci. Appl..

[4] Aitken D., Rivera D., Godoy-Faúndez A., Holzapfel E., Aitken D., Rivera D., Godoy-Faúndez A., Holzapfel E. (2016). Water Scarcity and the Impact of the Mining and Agricultural Sectors in Chile. Sustainability.

[5] Valdés-Pineda R., Pizarro R., García-Chevesich P., Valdés J.B., Olivares C., Vera M., Balocchi F., Pérez F., Vallejos C., Fuentes R. (2014). Water governance in Chile: Availability, management and climate change. J. Hydrol..

[6] Oyarzún J., Oyarzún R. (2011). Sustainable development threats, inter-sector conflicts and environmental policy requirements in the arid, mining rich, northern Chile territory. Sustain. Dev..

[7] Johnson D.B., Hallberg K.B. (2005). Acid mine drainage remediation options: A review. Sci. Total Environ..

[8] Obreque-Contreras J., Pérez-Flores D., Gutiérrez P., Chávez-Crooker P. (2015). Acid Mine Drainage in Chile: An Opportunity to Apply Bioremediation Technology. J. Waste Water Treat. Anal..

[9] Kefeni K.K., Msagati T.A.M., Mamba B.B. (2017). Acid mine drainage: Prevention, treatment options, and resource recovery: A review. J. Clean. Prod..

[10] Skousen J., Zipper C.E., Rose A., Ziemkiewicz P.F., Nairn R., McDonald L.M., Kleinmann R.L. (2017). Review of Passive Systems for Acid Mine Drainage Treatment. Mine Water Environ..

[11] Fu F., Wang Q. (2011). Removal of heavy metal ions from wastewaters: A review. J. Environ. Manag..

[12] Fernando W.A.M., Ilankoon I.M.S.K., Syed T.H., Yellishetty M. (2018). Challenges and opportunities in the removal of sulphate ions in contaminated mine water: A review. Miner. Eng..

[13] Westerhoff P., Alvarez P., Li Q., Gardea-Torresdey J., Zimmerman J. (2016). Overcoming implementation barriers for nanotechnology in drinking water treatment. Environ. Sci. Nano.

[14] Ren X., Chen C., Nagatsu M., Wang X. (2011). Carbon nanotubes as adsorbents in environmental pollution management: A review. Chem. Eng. J..

[15] Ihsanullah, Abbas A., Al-Amer A.M., Laoui T., Al-Marri M.J., Nasser M.S., Khraisheh M., Atieh M.A. (2016). Heavy metal removal from aqueous solution by advanced carbon nanotubes: Critical review of adsorption applications. Sep. Purif. Technol..

[16] Xu H., Ding M., Shen K., Cui J., Chen W. (2017). Removal of aluminum from drinking water treatment sludge using vacuum electrokinetic technology. Chemosphere.

[17] Upadhyayula V.K.K., Deng S., Mitchell M.C., Smith G.B. (2009). Application of carbon nanotube technology for removal of contaminants in drinking water: A review. Sci. Total Environ..

[18] Li Y.-H., Ding J., Luan Z., Di Z., Zhu Y., Xu C., Wu D., Wei B. (2003). Competitive adsorption of Pb^2+^, Cu^2+^ and Cd^2+^ ions from aqueous solutions by multiwalled carbon nanotubes. Carbon.

[19] Stafiej A., Pyrzynska K. (2007). Adsorption of heavy metal ions with carbon nanotubes. Sep. Purif. Technol..

[20] Tofighy M.A., Mohammadi T. (2011). Adsorption of divalent heavy metal ions from water using carbon nanotube sheets. J. Hazard. Mater..

[21] Fang Q., Chen B. (2012). Adsorption of perchlorate onto raw and oxidized carbon nanotubes in aqueous solution. Carbon.

[22] Vuković G.D., Marinković A.D., Škapin S.D., Ristić M.T., Aleksić R., Perić-Grujić A.A., Uskoković P.S. (2011). Removal of lead from water by amino modified multi-walled carbon nanotubes. Chem. Eng. J..

[23] Vuković G.D., Marinković A.D., Čolić M., Ristić M.D., Aleksić R., Perić-Grujić A.A., Uskoković P.S. (2010). Removal of cadmium from aqueous solutions by oxidized and ethylenediamine-functionalized multi-walled carbon nanotubes. Chem. Eng. J..

[24] Sun Y.-P., Fu K., Lin Y., Huang W. (2002). Functionalized Carbon Nanotubes: Properties and Applications. Acc. Chem. Res..

[25] Balasubramanian K., Burghard M. (2005). Chemically Functionalized Carbon Nanotubes. Small.

[26] Liao C., Zhao X.R., Jiang X.Y., Teng J., Yu J.G. (2020). Hydrothermal fabrication of novel three-dimensional graphene oxide-pentaerythritol composites with abundant oxygen-containing groups as efficient adsorbents. Microchem. J..

[27] Wang Z., Li X., Liang H., Ning J., Zhou Z., Li G. (2017). Equilibrium, kinetics and mechanism of Au^3+^, Pd^2+^ and Ag^+^ ions adsorption from aqueous solutions by graphene oxide functionalized persimmon tannin. Mater. Sci. Eng. C.

[28] He Y., Wu P., Xiao W., Li G., Yi J., He Y., Chen C., Ding P., Duan Y. (2019). Efficient removal of Pb(II) from aqueous solution by a novel ion imprinted magnetic biosorbent: Adsorption kinetics and mechanisms. PLoS ONE.

[29] Sahraei R., Sekhavat Pour Z., Ghaemy M. (2017). Novel magnetic bio-sorbent hydrogel beads based on modified gum tragacanth/graphene oxide: Removal of heavy metals and dyes from water. J. Clean. Prod..

[30] Rao G.P., Lu C., Su F. (2007). Sorption of divalent metal ions from aqueous solution by carbon nanotubes: A review. Sep. Purif. Technol..

[31] Lu C., Liu C. (2006). Removal of nickel(II) from aqueous solution by carbon nanotubes. J. Chem. Technol. Biotechnol..

[32] Ghaedi M., Montazerozohori M., Nazari E., Nejabat R. (2013). Functionalization of multiwalled carbon nanotubes for the solid-phase extraction of silver, cadmium, palladium, zinc, manganese and copper by flame atomic absorption spectrometry. Hum. Exp. Toxicol..

[33] Pashai Gatabi M., Milani Moghaddam H., Ghorbani M. (2016). Point of zero charge of maghemite decorated multiwalled carbon nanotubes fabricated by chemical precipitation method. J. Mol. Liq..

[34] Lee S., Zhang Z., Wang X., Pfefferle L.D., Haller G.L. (2011). Characterization of multi-walled carbon nanotubes catalyst supports by point of zero charge. Catal. Today.

[35] Pyrzyńska K., Bystrzejewski M. (2010). Comparative study of heavy metal ions sorption onto activated carbon, carbon nanotubes, and carbon-encapsulated magnetic nanoparticles. Colloids Surfaces A Physicochem. Eng. Asp..

[36] Li H., Dong X., da Silva E.B., de Oliveira L.M., Chen Y., Ma L.Q. (2017). Mechanisms of metal sorption by biochars: Biochar characteristics and modifications. Chemosphere.

[37] Babić B.M., Milonjić S.K., Polovina M.J., Kaludierović B.V. (1999). Point of zero charge and intrinsic equilibrium constants of activated carbon cloth. Carbon.

[38] Kodama S., Sekiguchi H. (2006). Estimation of point of zero charge for activated carbon treated with atmospheric pressure non-thermal oxygen plasmas. Thin Solid Films.

[39] Salam M.A., Makki M.S.I., Abdelaal M.Y.A. (2011). Preparation and characterization of multi-walled carbon nanotubes/chitosan nanocomposite and its application for the removal of heavy metals from aqueous solution. J. Alloys Compd..

[40] Wu C.H. (2007). Studies of the equilibrium and thermodynamics of the adsorption of Cu^2+^ onto as-produced and modified carbon nanotubes. J. Colloid Interface Sci..

[41] Li Y.-H., Luan Z., Xiao X., Zhou X., Xu C., Wu D., Wei B. (2003). Removal of Cu^2+^ Ions from Aqueous Solutions by Carbon Nanotubes. Adsorpt. Sci. Technol..

[42] Zhang J., Zou H., Qing Q., Yang Y., Li Q., Liu Z., Guo X., Du Z. (2003). Effect of chemical oxidation on the structure of single-walled carbon nanotubes. J. Phys. Chem. B.

[43] Datsyuk V., Kalyva M., Papagelis K., Parthenios J., Tasis D., Siokou A., Kallitsis I., Galiotis C. (2008). Chemical oxidation of multiwalled carbon nanotubes. Carbon.

[44] Jia Z., Wang Z., Liang J., Wei B., Wu D. (1999). Production of short multi-walled carbon nanotubes. Carbon.

[45] Bikiaris D., Vassiliou A., Chrissafis K., Paraskevopoulos K.M., Jannakoudakis A., Docoslis A. (2008). Effect of acid treated multi-walled carbon nanotubes on the mechanical, permeability, thermal properties and thermo-oxidative stability of isotactic polypropylene. Polym. Degrad. Stab..

[46] Hong C.E., Lee J.H., Kalappa P., Advani S.G. (2007). Effects of oxidative conditions on properties of multi-walled carbon nanotubes in polymer nanocomposites. Compos. Sci. Technol..

[47] Chiang Y.C., Lin W.H., Chang Y.C. (2011). The influence of treatment duration on multi-walled carbon nanotubes functionalized by H_2_SO_4_/HNO_3_ oxidation. Appl. Surf. Sci..

[48] Pavia D., Lampman G., Kriz G., Vyvyan J. (2008). Introduction to Spectroscopy.

[49] Kawai T., Miyamoto Y. (2008). Chirality-dependent C–C bond breaking of carbon nanotubes by cyclo-addition of oxygen molecule. Chem. Phys. Lett..

[50] Martínez M.T., Callejas M.A., Benito A.M., Cochet M., Seeger T., Ansón A., Schreiber J., Gordon C., Marhic C., Chauvet O. (2003). Sensitivity of single wall carbon nanotubes to oxidative processing: Structural modification, intercalation and functionalisation. Carbon.

[51] Ge Y., Li Z., Xiao D., Xiong P., Ye N. (2014). Sulfonated multi-walled carbon nanotubes for the removal of copper (II) from aqueous solutions. J. Ind. Eng. Chem..

[52] Li Y.H., Wang S., Luan Z., Ding J., Xu C., Wu D. (2003). Adsorption of cadmium(II) from aqueous solution by surface oxidized carbon nanotubes. Carbon.

[53] Gao Z., Bandosz T.J., Zhao Z., Han M., Qiu J. (2009). Investigation of factors affecting adsorption of transition metals on oxidized carbon nanotubes. J. Hazard. Mater..

[54] Bandosz T.J., Jagieƚƚo J., Schwarz J.A. (1993). Effect of Surface Chemical Groups on Energetic Heterogeneity of Activated Carbons. Langmuir.

[55] Perić J., Trgo M., Vukojević Medvidović N. (2004). Removal of zinc, copper and lead by natural zeolite-A comparison of adsorption isotherms. Water Res..

[56] Anastopoulos I., Hosseini-Bandegharaei A., Fu J., Mitropoulos A.C., Kyzas G.Z. (2018). Use of nanoparticles for dye adsorption: Review. J. Dispers. Sci. Technol..

[57] Mubarak N.M., Sahu J.N., Abdullah E.C., Jayakumar N.S. (2014). Removal of heavy metals from wastewater using carbon nanotubes. Sep. Purif. Rev..

[58] Ganesan P., Kamaraj R., Sozhan G., Vasudevan S. (2013). Oxidized multiwalled carbon nanotubes as adsorbent for the removal of manganese from aqueous solution. Environ. Sci. Pollut. Res..

[59] Cho H.-H., Wepasnick K., Smith B.A., Bangash F.K., Fairbrother D.H., Ball W.P. (2010). Sorption of Aqueous Zn[II] and Cd[II] by Multiwall Carbon Nanotubes: The Relative Roles of Oxygen-Containing Functional Groups and Graphenic Carbon. Langmuir.

[60] Lu C., Liu C., Su F. (2009). Sorption kinetics, thermodynamics and competition of Ni2+ from aqueous solutions onto surface oxidized carbon nanotubes. Desalination.

[61] Park J.H., Ok Y.S., Kim S.H., Cho J.S., Heo J.S., Delaune R.D., Seo D.C. (2016). Competitive adsorption of heavy metals onto sesame straw biochar in aqueous solutions. Chemosphere.

[62] Kosa S.A., Al-Zhrani G., Abdel Salam M. (2012). Removal of heavy metals from aqueous solutions by multi-walled carbon nanotubes modified with 8-hydroxyquinoline. Chem. Eng. J..

[63] Soltani H., Belmokhtar A., Zeggai F.Z., Benyoucef A., Bousalem S., Bachari K. (2019). Copper(II) Removal from Aqueous Solutions by PANI-Clay Hybrid Material: Fabrication, Characterization, Adsorption and Kinetics Study. J. Inorg. Organomet. Polym. Mater..

[64] Zhao X.R., Xu X., Teng J., Zhou N., Zhou Z., Jiang X.Y., Jiao F.P., Yu J.G. (2019). Three-dimensional porous graphene oxide-maize amylopectin composites with controllable pore-sizes and good adsorption-desorption properties: Facile fabrication and reutilization, and the adsorption mechanism. Ecotoxicol. Environ. Saf..

[65] Salam M.A., Al-Zhrani G., Kosa S.A. (2012). Simultaneous removal of copper(II), lead(II), zinc(II) and cadmium(II) from aqueous solutions by multi-walled carbon nanotubes. C. R. Chim..

[66] Zhang X., Huang Q., Liu M., Tian J., Zeng G., Li Z., Wang K., Zhang Q., Wan Q., Deng F. (2015). Preparation of amine functionalized carbon nanotubes via a bioinspired strategy and their application in Cu^2+^ removal. Appl. Surf. Sci..

[67] Elsehly E.M.I., Chechenin N.G., Bukunov K.A., Makunin A.V., Priselkova A.B., Vorobyeva E.A., Motaweh H.A. (2016). Removal of iron and manganese from aqueous solutions using carbon nanotube filters. Water Sci. Technol. Water Supply.

[68] Jiang L., Li S., Yu H., Zou Z., Hou X., Shen F., Li C., Yao X. (2016). Amino and thiol modified magnetic multi-walled carbon nanotubes for the simultaneous removal of lead, zinc, and phenol from aqueous solutions. Appl. Surf. Sci..

[69] Kuo C.-Y., Wu C.-H., Wu J.-Y. (2008). Adsorption of direct dyes from aqueous solutions by carbon nanotubes: Determination of equilibrium, kinetics and thermodynamics parameters. J. Colloid Interface Sci..

[70] Li Y.H., Di Z., Ding J., Wu D., Luan Z., Zhu Y. (2005). Adsorption thermodynamic, kinetic and desorption studies of Pb^2+^ on carbon nanotubes. Water Res..

[71] Simonin J.P. (2016). On the comparison of pseudo-first order and pseudo-second order rate laws in the modeling of adsorption kinetics. Chem. Eng. J..

[72] Liang P., Liu Y., Guo L., Zeng J., Lu H. (2004). Multiwalled carbon nanotubes as solid-phase extraction adsorbent for the preconcentration of trace metal ions and their determination by inductively coupled plasma atomic emission spectrometry. J. Anal. At. Spectrom..

[73] Lu C., Chiu H., Liu C. (2006). Removal of Zinc(II) from Aqueous Solution by Purified Carbon Nanotubes: Kinetics and Equilibrium Studies. Ind. Eng. Chem. Res..

[74] Yan X.M., Shi B.Y., Lu J.J., Feng C.H., Wang D.S., Tang H.X. (2008). Adsorption and desorption of atrazine on carbon nanotubes. J. Colloid Interface Sci..

[75] Koochaki-Mohammadpour S.M.A., Torab-Mostaedi M., Talebizadeh-Rafsanjani A., Naderi-Behdani F. (2014). Adsorption Isotherm, Kinetic, Thermodynamic, and Desorption Studies of Lanthanum and Dysprosium on Oxidized Multiwalled Carbon Nanotubes. J. Dispers. Sci. Technol..

[76] Pu Y., Yang X., Zheng H., Wang D., Su Y., He J. (2013). Adsorption and desorption of thallium(I) on multiwalled carbon nanotubes. Chem. Eng. J..

[77] Liang S., Li G., Tian R. (2016). Multi-walled carbon nanotubes functionalized with a ultrahigh fraction of carboxyl and hydroxyl groups by ultrasound-assisted oxidation. J. Mater. Sci..

[78] Xia W., Wang Y., Bergsträßer R., Kundu S., Muhler M. (2007). Surface characterization of oxygen-functionalized multi-walled carbon nanotubes by high-resolution X-ray photoelectron spectroscopy and temperature-programmed desorption. Appl. Surf. Sci..

[79] Mazov I., Kuznetsov V.L., Simonova I.A., Stadnichenko A.I., Ishchenko A.V., Romanenko A.I., Tkachev E.N., Anikeeva O.B. (2012). Oxidation behavior of multiwall carbon nanotubes with different diameters and morphology. Appl. Surf. Sci..

[80] Gómez S., Rendtorff N.M., Aglietti E.F., Sakka Y., Suárez G. (2016). Surface modification of multiwall carbon nanotubes by sulfonitric treatment. Appl. Surf. Sci..

[81] Fan Q., Sun J., Chu L., Cui L., Quan G., Yan J., Hussain Q., Iqbal M. (2018). Effects of chemical oxidation on surface oxygen-containing functional groups and adsorption behavior of biochar. Chemosphere.

[82] Yang J.-Y., Yue B.-Y., Jie-Teng, Liu Q., Jiang X.-Y., Zhong M., Zhou F.-L., Yu J.-G. (2019). Dichlorocarbene modified graphene oxide nanocomposite fabricated by a facile hydrothermal method and its adsorption properties toward rare earth elements. Desalin. Water Treat..

[83] Zhao X.-R., Xu X., Jiang X.-Y., Jie-Teng, Yu J.-G. (2019). Facile fabrication of three-dimensional and recyclable graphene oxide-melamine composites with high removal efficiency. Desalin. Water Treat..

[84] Sahai A., Goswami N., Kaushik S.D., Tripathi S. (2016). Cu/Cu_2_O/CuO nanoparticles: Novel synthesis by exploding wire technique and extensive characterization. Appl. Surf. Sci..

[85] Gan Z.H., Yu G.Q., Tay B.K., Tan C.M., Zhao Z.W., Fu Y.Q. (2004). Preparation and characterization of copper oxide thin films deposited by filtered cathodic vacuum arc. J. Phys. D Appl. Phys..

[86] Liu Y., Liao L., Li J., Pan C. (2007). From Copper Nanocrystalline to CuO Nanoneedle Array:  Synthesis, Growth Mechanism, and Properties. J. Phys. Chem. C.

[87] Biesinger M.C. (2017). Advanced analysis of copper X-ray photoelectron spectra. Surf. Interface Anal..

[88] Biesinger M.C., Payne B.P., Grosvenor A.P., Lau L.W.M., Gerson A.R., Smart R.S.C. (2011). Resolving surface chemical states in XPS analysis of first row transition metals, oxides and hydroxides: Cr, Mn, Fe, Co and Ni. Appl. Surf. Sci..

[89] Ilton E.S., Post J.E., Heaney P.J., Ling F.T., Kerisit S.N. (2016). XPS determination of Mn oxidation states in Mn (hydr)oxides. Appl. Surf. Sci..

[90] Jiang J., Kucernak A. (2002). Electrochemical supercapacitor material based on manganese oxide: Preparation and characterization. Electrochim. Acta.

[91] Gogurla N., Sinha A.K., Santra S., Manna S., Ray S.K. (2014). Multifunctional Au-ZnO plasmonic nanostructures for enhanced UV photodetector and room temperature NO sensing devices. Sci. Rep..

[92] Kothandapani J., Ganesan A., Mani G.K., Kulandaisamy A.J., Rayappan J.B.B., Selva Ganesan S. (2016). Zinc oxide surface: A versatile nanoplatform for solvent-free synthesis of diverse isatin derivatives. Tetrahedron Lett..

[93] Byrne R.H., Kump L.R., Cantrell K.J. (1988). The influence of temperature and pH on trace metal speciation in seawater. Mar. Chem..

[94] Spurgeon D.J., Lofts S., Hankard P.K., Toal M., McLellan D., Fishwick S., Svendsen C. (2006). Effect of pH on metal speciation and resulting metal uptake and toxicity for earthworms. Environ. Toxicol. Chem..

[95] Albrecht T., Addai-Mensah J., Fornasiero D. (2011). Effect of pH, Concentration and Temperature on Copper and Zinc Hydroxide Formation/Precipitation in Solution. https://pdfs.semanticscholar.org/6ae0/a349aea2ddfb5d1fcd34ff92bff180d5b15f.pdf.

[96] Cuppett J.D., Duncan S.E., Dietrich A.M. (2006). Evaluation of Copper Speciation and Water Quality Factors That Affect Aqueous Copper Tasting Response. Chem. Senses.

[97] Takeno N. (2005). Atlas of Eh-pH diagrams. Geol. Surv. Japan Open File Rep..

[98] Kandah M.I., Meunier J.L. (2007). Removal of nickel ions from water by multi-walled carbon nanotubes. J. Hazard. Mater..

[99] Hadavifar M., Bahramifar N., Younesi H., Li Q. (2014). Adsorption of mercury ions from synthetic and real wastewater aqueous solution by functionalized multi-walled carbon nanotube with both amino and thiolated groups. Chem. Eng. J..

[100] Heidari A., Younesi H., Mehraban Z. (2009). Removal of Ni(II), Cd(II), and Pb(II) from a ternary aqueous solution by amino functionalized mesoporous and nano mesoporous silica. Chem. Eng. J..

